# Advances in the Applications of Clinoptilolite-Rich Tuffs

**DOI:** 10.3390/ma17061306

**Published:** 2024-03-12

**Authors:** Jelena Pavlović, Jasna Hrenović, Dragan Povrenović, Nevenka Rajić

**Affiliations:** 1 Institute of Soil Science, Teodora Drajzera 7, 11000 Belgrade, Serbia; soils.pavlovic@gmail.com; 2 Faculty of Science, Department of Biology, University of Zagreb, 10000 Zagreb, Croatia; jasna.hrenovic@biol.pmf.hr; 3 Faculty of Technology and Metallurgy, University of Belgrade, Karnegijeva 4, 11120 Belgrade, Serbia; povrenovic@tmf.bg.ac.rs; 4 Faculty of Ecology and Environmental Protection, University “Union—Nikola Tesla”, Cara Dušana 62–64, 11158 Belgrade, Serbia

**Keywords:** natural zeolite, adsorption, catalysis, antimicrobial activity, nano oxides, biomass

## Abstract

Adsorptive, catalytic, and antibacterial properties of clinoptilolite-rich tuffs (ZT) are presented here. ZT transformed into Fe-containing ZT (Fe-ZT) removes various organic and inorganic anions from water. Fe-ZT, which contains selenium, is beneficial for growing *Pleurotus ostreatus* mushrooms. The fungi convert inorganic Se from Fe-ZT into a more useful organically bonded form. ZT and Fe-ZT as supplements retain nitrogen and potassium in sandy, silty loam and silty clay soils. ZT shows an affinity toward toxic metal cations, which are essential for cleaning contaminated water. The adsorption of atenolol, acetylsalicylic, and salicylic acid onto M-ZT (M–Cu^2+^, Mn^2+^, Ni^2+^, or Zn^2+^) from water solutions suggests that both the natures of M and pharmaceuticals have a significant impact on the adsorption mechanism and determine the adsorption capability of the ZT. ZT is an excellent carrier for ultrafine (2–5 nm) nano oxide particles, which have been shown to have catalytic activity in different chemical processes and photodegradation reactions of organic pollutants. ZT can also be transformed into SO_4_-SnO_2_-ZT, which is catalytically active as a solid acid. M-ZT is an effective carrier of valuable bacteria. Ag-ZT possesses beneficial bactericidal activity in disinfecting water and soil remediation.

## 1. Introduction

Zeolites are crystalline porous aluminosilicates with a three-dimensional lattice structure built from a network of corner-sharing tetrahedra, TO_4_ (T= Si, Al), which contain Si and Al atoms in the center of tetrahedra. Only species with the proper size may diffuse through the lattice owing to the crystal structure’s geometrically precise structured channels and cages. As a result, zeolites can be employed as molecular sieves and in ion-exchange, -adsorption, and -separation processes. Zeolites can also be converted into strong solid acids and used as heterogeneous catalysts [1,2,3].

The most common type of natural zeolite is clinoptilolite. Eight- and ten-member ring channels with diameters of up to 0.7 nm reveal an easily accessible open reticular structure [4,5]. Two channels run parallel to the c-axis. The A channels comprise tightly compressed ten-membered rings with an aperture of 0.31 × 0.75 nm, and the B channels comprise eight-membered rings with an aperture of 0.36 × 0.46 nm. The C channels have an aperture of 0.28 × 0.47 nm and are formed by eight-membered rings parallel to the a-axis. Water molecules and exchangeable alkali and alkali earth cations occupy crystallographically specific extra-framework sites. Clinoptilolite samples from different regions have different ion-exchange abilities due to variances in the Si/Al molar ratio and the types of extra-framework cations.

Clinoptilolite-rich deposits are primarily found in regions that have experienced hydrothermal processes and volcanic activity. Europe’s principal deposits of clinoptilolite-rich tuffs are in Slovakia, Ukraine, Turkey, Italy, and Romania. Typically, the zeolite minerals coexist in the specific rock. Certain minor and accessory minerals, including feldspar, the mica group, chlorite, cristobalite, zircon, iron, sulfur, and clay minerals, are linked to zeolite mineralization. The components of zeolite mineralization are regarded as undesirable impurities [6].

In recent decades, the adsorption, catalytic, and antibacterial characteristics of clinoptilolite-rich tuffs (ZT) from two deposits, Vranjska Banja and Slanci (Serbia), containing up to 80 wt.% clinoptilolite, with quartz and feldspar as the primary mineral impurities, have been extensively studied [7,8,9,10,11,12,13,14,15,16,17,18,19,20,21,22,23,24,25,26,27,28,29,30,31,32,33,34,35,36,37,38]. This review compares these results with ZTs from other regions. It is important to note that the high degree of grain size, porosity, cation-exchange capacity, clinoptilolite chemical composition, and experimental conditions all affect the properties under study. As a result, comparisons are frequently challenging to make and may produce conclusions that are not sufficiently trustworthy.

The clinoptilolite content of the ZT from deposits in Serbia is similar to that of the clinoptilolite-rich tuff from Ukraine, including roughly 70 wt.% [6], the tuff from Slovakia containing approximately 85 wt.% clinoptilolite [39], the tuff from Iran containing 82 wt.%, and the tuff from Turkey containing 76 wt.%. Clinoptilolite from Serbia has a Cation-Exchange Capacity (CEC) of 170–180 mmol M^+^/100 g, while clinoptilolite from Ukraine has a CEC of 64 mmol M^+^/100 g [39], clinoptilolite from has a CEC of 130 mmol M^+^/100 g, and clinoptilolite from Iran has a CEC of 126 mmol M^+^/100 g [16]. The variation in the Si/Al molar ratio and the type of ion-exchangeable cations can be attributed to the variation in CEC value.

## 2. Conversion of ZT

### 2.1. Conversion of ZT to Fe-Containing ZTs

Because Fe-oxyhydroxides, which are available commercially, are usually used to remove toxic As from subterranean water, which is not cost-effective, upgrading ZT to Fe-ZT or Fe_3_O_4_-ZT makes it an economically viable adsorbent [40,41,42,43,44,45]. A straightforward two-step process has typically been employed. Treatment of ZT with a water solution of Fe(NO_3_)_3_ in an acetate buffer (pH = 3.6) followed by adding NaOH until pH = 7 and heating of the product at 80 °C to a constant mass gives Fe-ZT. EDS analysis of Fe-ZT revealed that the content of exchangeable cations significantly decreased relative to ZT and that the content of Fe was significantly higher than the decrease in the ZT-exchangeable cations. This indicates that the conversion of ZT to Fe-ZT includes at least two processes: (1) ion exchange and (2) precipitation of the Fe(III) species at the ZT surface. The conversion of ZT to Fe_3_O_4_-ZT can be performed in a water solution of FeCl_3_·6H_2_O and FeSO_4_ in a molar ratio of 2:1 under N_2_ [17,46,47,48,49].

**Figure 1:**
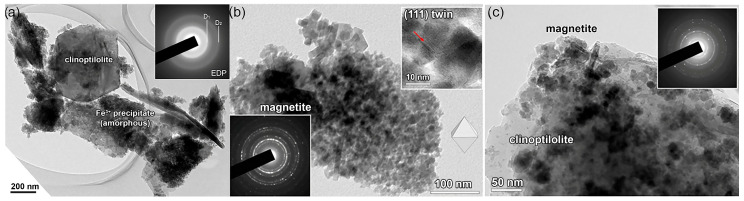
TEM images of (**a**) Fe-ZT: bright-field image of clinoptilolite sheets coated with a precipitate rich in Fe(III). The amorphous nature of the Fe(III) precipitate is confirmed by its electron diffraction pattern (EDP) in the upper right corner. (**b**) Nano oxide particles of magnetite: EDP is shown in the lower left corner. The upper right corner shows characteristic magnetite octahedral morphology with many crystals connected by the {111} spinel-twin law that forms through self-assembly. (**c**) Fe_3_O_4_-ZT: the EDP of magnetite layers on clinoptilolite sheets is given in the upper right corner [19].

The treatments led to an increase in Fe content from 0.21 (ZT) to 18.1 wt.% for Fe-ZT in the clinoptilolite from deposits in Serbia [19], whereas, from Slovakia, the Fe content was lower [50] increased from 1.03 (ZT) to 5.82 wt.% (Fe-ZT) under similar conditions. For Fe_3_O_4_-ZT, the increase in Fe was 5.63 wt.%. It was proposed that Fe species precipitation would occur at the ZT because the EDS analysis revealed that the Fe content is higher than predicted based on the CEC value. TEM analyses verified it (Figure 1).

Accumulating the Fe-containing precipitates increases the specific surface area of ZT from 28.6 to 140.3 m^2^ g^−1^ for Fe-ZT and 45.2 m^2^ g^−1^ for Fe_3_O_4_-ZT. Amorphous precipitate in Fe-ZT significantly increases the specific surface area of ZT, whereas in Fe_3_O_4_-ZT, the well’s crystalline magnetite nanoparticles in the range of 5–30 nm cover the surface of ZT [17,19]. Similarly, Fe-ZT obtained from ZT from the deposit Donje Jesenje (Croatia) was found to have a specific surface area around three times larger than that of parent ZT [40]. Furthermore, a marginally greater rise in specific surface area has been noted for Fe_3_O_4_-ZT, synthesized from ZT originating from a deposit from Turkey [47].

It seems likely that the increase in the specific surface of Fe-ZT can be attributed to forming a second porous system at the ZT surface. The modification of ZT to Fe_3_O_4_–ZT increased the specific surface area to a lower extent but had two additional effects on ZT: (a) a partial dealumination (the Si/Al ratio increased from 5.0 to 6.1) and (b) magnetic properties. Powder XRD analysis showed that the partial dealumination did not significantly affect the crystallinity of ZT [17]. Most importantly, magnetite on the ZT surface introduced magnetism to ZT. In magnetic fields lower than 1.5 T, pure magnetite and Fe_3_O_4_-ZT exhibit comparable magnetic behavior with saturation magnetization values of 49.57 and 8.93 emu g^−1^, respectively. The lower saturation magnetization value of Fe_3_O_4_-ZT was ascribed to the precipitation of magnetite on the ZT surface. The important notice is the fact that Fe_3_O_4_-ZT maintains its magnetic characteristics during the adsorption process. This lets the spent adsorbent be magnetically separated from water media [17].

Recently, a report was published on the green synthesis of clinoptilolite Fe_3_O_4_-zeolite nanocomposite using leaf extract from *Laurus nobilis* L. [51]. The composite displays characteristics of superparamagnetic properties. A novel solid-phase microextraction technique was created to extract and determine Rhodamine B utilizing high-performance liquid chromatography and clinoptilolite zeolite-coated magnetic nanocomposite.

### 2.2. Formation of Ultrafine Nano Oxide Particles inside ZT

**Figure 2:**
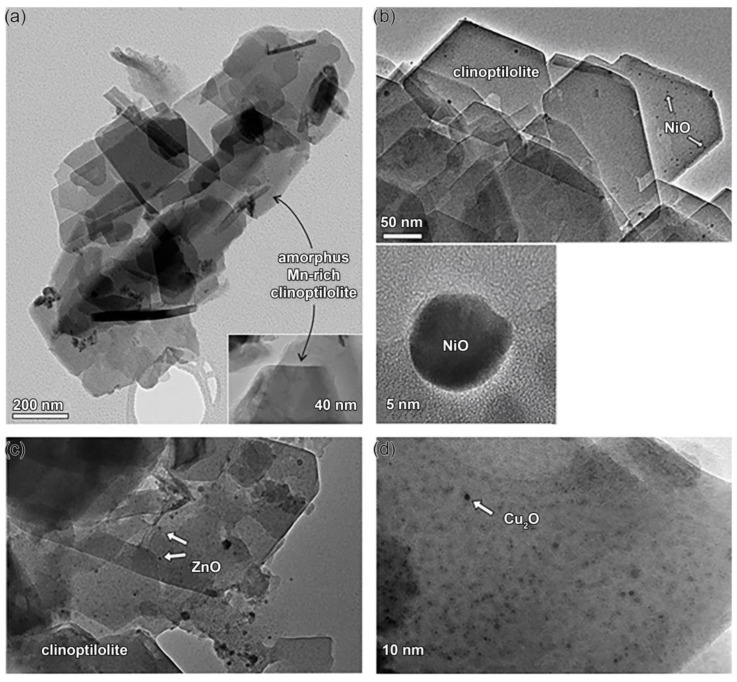
TEM image of the calcined M-ZT: (**a**) high-magnification image (bottom right) shows a uniform surface of an amorphous clinoptilolite grain without any visible oxide nanoparticles; (**b**) Ni-ZT grains with NiO nanoparticles. A single NiO particle and SAED pattern recorded across the NiO particles are shown below: (**c**) Zn-ZT grains with ZnO nanoparticles and (**d**) the surface of a Cu-ZT grain with multiple Cu_2_O nanoparticles [9].

Many nano oxide particles are effective catalysts in various chemical processes. However, most often, they must be supported by a suitable material that offers exposed surface area and mechanical strength because of their small size. With both of the desired qualities, clinoptilolite might be a promising option. Thus, the treatment of ZT with MCl_2_ aqueous solutions (M = Ni, Cu, Mn, Zn) gave M-containing products (Ni-ZT, Cu-ZT, Mn-ZT, and Zn-ZT), which, after drying to a constant mass and calcination at 550 °C, produced nano oxide particles at the clinoptilolite surface [9,12]. EDS analyses showed that Na^+^ ions are replaced in the Na-ZT by Mn(II), Ni(II), Cu(II), and Zn(II). The degree of ion exchange is different and depends on the chemical nature of the cations in aqueous solution: Ni(II) has the least amount of ion exchange (8%); Mn(II) and Zn(II) replace roughly 21% of the Na^+^ ions, while for Cu(II), the exchange degree is the highest at 38%. According to a recent report, cations prefer four crystallographic positions in the clinoptilolite channels depending on their chemical nature [52]. Mn^2+^ prefers the cation site inside channel B (the site M2), Ni^2+^ is located primarily at two sites in the A channel (M1, M3: M1 is positioned in the center, and M3 is at the entrance to channel C), and Zn^2+^ occupies all three different sites inside three channels [53]. The differences in cation’s accessibility to the extra-framework ion-exchange sites explain the differences observed during the calcination and dehydration of M-ZT. According to SEAD, the dehydrated Mn-ZT has no extra oxide phase compared to Ni-ZT, Cu-ZT, and Zn-ZT. As shown in Figure 2, NiO, ZnO, and Cu_2_O oxide nanoparticles are randomly distributed in the clinoptilolite matrix. The last three solids contain crystal clusters that are populated with spherical nanocrystalline particles to varying degrees, which are oxides of Ni(II), Zn(II), and Cu(I) [9].

The average particle size of the NiO crystallites is approximately 5 nm in diameter, and their sizes range from 2 to 7 nm. SEAD pattern revealed that they belong to the cubic NiO structure (JCPDF # 78-0643). It is implied by the lack of preferential faceting of the cubic NiO crystals that a rapid crystallization occurred during the calcination. Similarly, the SAED identified a polycrystalline ZnO with a wurtzite structure in the calcined Zn-ZT (JCPDS 00-003-0888). The Cu_2_O (cuprite) nanoparticles crystallized due to the calcination of the Cu-ZT. In this case, the reduction of Cu(II) to Cu(I) occurred most probably due to the higher lattice enthalpy of the Cu_2_O. The estimated average size of cuprite particles was only 2 nm.

Nano-sized MnxOy-containing clinoptilolite has been synthesized using a hydrothermal method in a temperature range of 80–180 °C. Manganese found in three oxidation states, Mn^2+^ (37.8%), Mn^3+^ (14.2%), and Mn^4+^ (48%), explained the high catalytic activity of the composite [54].

The small pore size of the clinoptilolite lattice, the particular channel arrangement, and the crystallographic sites occupied by particular cations all appear to impact the crystallization of oxide phases during the calcination process. The steric restriction imposed by the clinoptilolite lattice enables the uniform distribution of the ultrafine nanoparticles, and the clinoptilolite lattice itself becomes a very good carrier of nanoparticles.

### 2.3. Conversion of ZT into SnO_2_-ZT and SO_4_-SnO_2_-ZT

The main benefits of solid acids and super acids over ordinary mineral acids or Lewis acids are that they do not need as many toxic or corrosive reagents, nor do they have handling, post-reaction separation, recovery, recycling, or contamination issues. Some metal oxides, such as titanium, zirconia, and SnO_2_, have become highly effective solid acid catalysts because of their super-acidity, high activity, and selectivity. Sulfated SnO_2_ has recently been shown to have greater acid strength and catalytic activity than other sulfated metal oxides [55,56]. With this in mind, the immobilization of SO_4_-SnO_2_ onto ZT was studied to prevent possible sulfate leaching reported for SO_4_-SnO_2_ [18].

ZT was converted into SO_4_-SnO_2_-ZT using a three-step procedure: (1) conversion of ZT to H-ZT by treating ZT with 1 mol dm^−3^ HCl at 100 °C and then with 0.2 mol dm^−3^ NH_4_OH at 65 °C; (2) preparation of the SnO_2_-containing ZT (SnO_2_-ZT) using an ethanolic solution of SnCl_2_ (*C*_0_ = 2 g dm^−3^) and NH_4_OH solution followed by calcination at 400 °C; and (3) sulfation of SnO_2_-ZT using (NH_4_)_2_SO_4_ followed by calcination at 400 °C to obtain SO_4_-SnO_2_-ZT [18].

In the first step of the procedure, ZT was converted into its hydrogen form, H-ZT. The treatment does not affect clinoptilolite’s crystallinity, but the lattice underwent a partial dealumination, as evidenced by the increase in the Si/Al molar ratio from 4.9 to 7. The content of Na drastically decreased, and K, Ca, and Mg were not found, which confirms that ZT is essentially transformed into H-ZT. Subsequent transformation of H-ZT into SnO_2_-ZT includes the treatment of H-ZT in a Sn(II) solution in an alkaline medium, resulting in a minor reduction of crystallinity, which is not affected by further transformation of SnO_2_-ZT into SO_4_-SnO_2_-ZT. The solid-state Al27 NMR evidences a partial dealumination of ZT. Besides the peak characteristic for Al atoms located in tetrahedral positions of the zeolite framework (AlO_4_ structural units) at 55 pm, maxima corresponding to octahedrally coordinated extra-framework Al species (0 ppm), as well as to the extra-framework five-coordinated Al (30 ppm), appear in the spectra. Moreover, the ^29^Si MAS NMR spectra display peaks connected to various Si environments and are assigned to the Si atoms with H atoms in their vicinity.

The presence of SnO_2_ in SnO_2_-ZT was verified by X-ray photoelectron spectroscopy (XPS). According to the XPS depth profiles, the Sn concentration decreases from the top to the bottom of the sample. There is a slight accumulation of Sn at the surface, which is more noticeable in the samples with a higher Sn content (1.6–2.4 at.%). The unexpected result was the observation of Sn within the clinoptilolite lattice, indicating that the Sn species are present both inside the lattice and on the zeolite’s surface. It is proposed that holes are formed in the lattice via a dealumination modification. Moreover, covalent Si–O–Sn bonds are suggested at the Sn(IV) species in the holes.

The last modification step is the sulfation of SnO_2_-ZT to SO_4_-SnO_2_-ZT, including treatment with (NH_4_)_2_SO_4_. This brings about a partial pore blockage of the clinoptilolite lattice, evidenced by a 20% decrease in the specific surface area for the sample with the highest Sn amount.

The purpose of this conversion of SnO_2_-ZT to SO_4_-SnO_2_-ZT was to give the ZT an acidic quality that would be potent enough to catalyze the esterification of levulinic acid and thus replace the highly corrosive H_2_SO_4_ that is typically used in this process. An FTIR spectra analysis of adsorbed pyridine was used to investigate the acidity of SnO_2_-ZT and SO_4_-SnO_2_-ZT. The SnO_2_-ZT spectra contain both Lewis and Brönsted acid sites. There are significantly more Lewis acid sites than Brönsted sites due to the contribution of coordinated unsaturated Sn ions from the Sn(IV) species inside the lattice. The role of Sn(IV) in the creation of Lewis acid sites is supported by the fact that the number of Brönsted acid sites does not change significantly with the Sn content while the number of Lewis acid sites increases. Finally, the amounts of Brönsted and Lewis acid sites are significantly higher for sulfated samples, indicating that sulfate species increase the Brönsted acid strength of the hydroxyl groups on the surface of SnO_2_ particles and function as Lewis acid sites.

## 3. Adsorption Studies

### 3.1. M(II) Adsorption (M–Mg, Mn, Ni, Cu, Zn, Pb) onto Na-ZT

Water and wastewater have been treated with ZTs, and this method continues to show promise for environmental cleaning processes. Over the past few decades, the application of ZTs has focused on eliminating ammonium and heavy metals due to ZT’s pronounced ion-exchange capabilities [57,58,59,60,61,62,63,64]. Here, ZT’s affinity (from deposits in Serbia) is presented for some metal cations. The experiments were conducted at 25–55 °C with an initial M(II) concentration of 1.5–6.0 mmol M dm^−3^, using a batch method, and using the (solution volume)/(solid weight) ratio 100 cm^3^: Na-ZT (ZT enriched with Na^+^ by ion-exchange pretreatment) removes the studied cations from water media via an endothermic ion-exchange reaction. Specifically, metal ions from an aqueous solution that come into contact with the ZT replace sodium ions from the clinoptilolite lattice. It is not found that intra-particle diffusion is the rate-limiting step in the ion-exchange process, even though all of the metal ions under study have hydrated radii that are noticeably larger than the clinoptilolite lattice aperture. It can be proposed that the coordination sphere of the cations alters during the reaction. Table 1 gives the removal efficiency of the Na-ZT.

For all studied cations, the removal efficiency rises with temperature. As a result, the removal efficiency toward Cu(II) increased by roughly 14% when the temperature increased from 25 to 45 °C. Moreover, the pseudo-second-order rate model gives the most accurate description of adsorption kinetics for each of the cations. However, the rate of adsorption increases with temperature only for Mn^2+^ and Ni^2+^; the increase is significant only for Mn^2+^. This can be due to the influence of the hydrolysis of metal ions on the adsorption kinetics [10]. The outcomes of the detailed crystal-structure analyses of Pb-exchanged zeolites provide a potential explanation. Research revealed that a change in the coordination sphere of Pb(II) ions occurs concurrently with the ion exchange of Pb(II) in zeolite lattices [65]. The last modification makes the current Pb(II) ion species smaller, facilitating a simple exchange of Na^+^ for Pb^2+^ within the zeolite channels. Because of the increased hydrolysis at higher temperatures, the formation of polynuclear metal species is likely. The large polynuclear hydrolysis products are less able to fit through the zeolite pore system due to their larger size, which explains the irregularities in the rate change by temperature. At 25 °C, the rate increases in the series Mn^2+^ < Zn^2+^ < Pb^2+^ < Ni^2+^< Cu^2+^ ≈ Mg^2+^, whereas at 55 °C, the rate of adsorption is the highest for Mn^2+^ [8,10,11,20,21,22,23].

### 3.2. Adsorption of Nitrate and Phosphate onto Fe-ZT

Both nitrogen and phosphorus are essential elements found in a variety of sectors, including agriculture and various industries. But phosphate and nitrate ions are also contaminants. The primary cause of eutrophication in lakes, reservoirs, and rivers is an overabundance of these anions [66,67,68]. Globally, elevated concentrations of these ions during the last ten years have sparked grave worries. As a result, effective techniques for removing them from water are required to protect water supplies.

The negatively charged zeolite lattice reduces phosphate and nitrate adsorption efficiency by clinoptilolite; therefore, zeolite surface modification, such as coating with cationic surfactants [68,69,70,71], is applied to enable zeolite removal efficiency. Recent research, however, highlights the toxicity of cationic surfactants and advises using them with care [72,73]. Considering this, modified zeolite forms that employ non-compromising materials for modification are investigated. Fe-ZT was shown to be a suitable adsorbent for oxoanionic species [40,41,42,43,44,45,74].

Solutions of KNO_3_ and KH_2_PO_4_ (1–6 mmol NO_3_^−^/PO_4_^3–^ dm^−3^) were used for the nitrate and phosphate adsorption by Fe-ZT [19,23]. The equilibrium adsorption data for both anions agree with the Langmuir isotherm, giving the Langmuir constant (*R_L_*) values in the range of 0–1, which is characteristic of favorable adsorption.

The adsorption mechanism is explained by electrostatic interactions between negatively charged nitrate and phosphate anions and positively charged M–OH_2_^+^ groups on the metal-hydroxy-containing Fe-ZT. At lower pH, the M–OH_2_^+^ groups arise due to the reaction between the hydronium ions from the solution and the surface hydroxyl groups present on Fe-ZT. However, nitrate ions may also be partially bound through ion exchange, whereby they replace hydroxyl ions [24].

The highest affinity toward phosphate ions Fe-ZT was shown at pH = 6.5. Removal efficiency increases with temperature, and the adsorption is described well by the Langmuir isotherm. Adsorption kinetics follows the Lagergren pseudo-second-order model. However, the ^31^P NMR study showed that the adsorption mechanism of phosphate is complex, including electrostatic interaction and more prominent covalent bonding between phosphate ion and Fe(III). The phosphate is predominantly bonded as a bidentate ligand [19].

### 3.3. Adsorption of Ciprofloxacin onto Fe_3_O_4_-ZT

Adsorption of ciprofloxacin (CIP) was studied for the initial CIP concentrations in the range of 0.04 to 0.2 mmol dm^−3^ [17]. The adsorption experiments were conducted at pH ~5, where CIP is present as the CIP^+^ ion. Fe_3_O_4_-ZT was an excellent adsorbent in the 15 to 30 °C temperature range. The adsorption is very fast, so more than 80% of the maximum adsorption capacity is reached within the first 10 min. Adsorption kinetics follows the Lagergren pseudo-second-order equation, and the Langmuir isotherm model describes the equilibrium adsorption data. Because the adsorbed CIP could not be removed by different treatments, including the acid-base treatment and ion exchange, it seems likely that the adsorption mechanism involves very strong electrostatic interactions between the negatively charged Fe_3_O_4_-ZT surface and the cationic form of CIP.

The presence of nano-magnetite particles on ZT brings magnetism to ZT, which allows the easy separation of the saturated adsorbent by simple magnetic separation. Moreover, it is worth noticing that the CIP-containing Fe_3_O_4_-ZT exhibits strong antibacterial activity toward pathogens (*E. coli and S. aureus*), suggesting its possible application in water disinfection [17].

The regeneration of the CIP-containing Fe_3_O_4_-ZT is successfully achieved by a plasma treatment operating at atmospheric pressure with air as the working gas [24]. Non-thermal plasma does not affect the zeolite crystal structure nor its textural properties, suggesting that the treatment can be a convenient method for regenerating the mineral adsorbent.

Rouhani et al. [49] have also verified the adsorption efficiency of Fe_3_O_4_-ZT toward antibiotics. Tetracycline was removed with 98.6% efficiency under optimal reaction conditions, with pH = 7–8 yielding the maximum removal efficiency. The physical adsorption mechanisms included tetracycline polar molecules’ van der Waals forces and hydrogen bonds with Fe_3_O_4_-ZT functional groups.

### 3.4. Adsorption of Atenolol, Acetylsalicylic Acid, and Salicylic Acid onto M-ZT

ZT and M-ZT (M–Mn(II), Ni(II), Cu(II), Zn) were tested for their adsorption capacities toward three pharmaceuticals that are known to be emerging water contaminants: atenolol (ATL), a beta-blocker drug, acetylsalicylic acid (ASA), a non-steroidal anti-inflammatory drug commonly used in organic synthesis, and salicylic acid (SA), widely used in organic synthesis [26].

The studies were carried out microcalorimetrically. The profiles of the heat flow signals are completed after 30 min, showing that the adsorption reached equilibrium. Adsorption isotherms differ for the same type of pharmaceutical, suggesting that the chemical nature of M influences the adsorption process. Also, different shapes of adsorption isotherms and different adsorption capacities are obtained for the different M-ZTs.

ATL adsorption capacities are found: Ni-ZT displays the highest adsorption capacity with multilayer adsorption. The final surface concentrations of ATL are from 40 to 115 μmol g^−1^. Cu-ZT displayed the lowest adsorption abilities. A decrease in the differential heat (*Q*_diff_) indicates that ATL molecules interact with heterogeneous solid surfaces: a drop from 40 kJ mol^−1^ to 10 kJ mol^−1^ is evident for Cu- and Ni-ZT and from 35 to 10 kJ mol^−1^ for Zn- and Mn-ZT. Initial *Q*_diff_ values suggest that a fraction of ATL molecules strongly interact with active sites, whereas most ATL molecules are physisorbed on M-ZTs.

In the case of SA, different adsorbed amounts and equilibrium concentrations were found for ZT and M-ZTs. The isotherm shapes differ significantly: the isotherms for Ni-Z and Mn-Z indicate multilayer adsorption, whereas the isotherms of SA on ZT, Cu-Z, and Zn-Z show saturation. The final surface concentrations of SA (amounts adsorbed per gram of solid adsorbent) are from 5 to 15 μmol g^−1^. The values obtained for the SA adsorption show a strong interaction with M-ZT for at least a fraction of the salicylic acid molecules, as they are significantly higher than those found for ATL. For the Ni-, Cu-, Zn-, and Mn-ZT, the corresponding initial differential heat values for the SA adsorption are 110, 109, 105, and 55 kJ mol^−1^. The initial *Q*_diff_ for SA on ZT is significantly lower (21 kJ mol^−1^), indicating that ZT has a considerably lower affinity toward SA than M-ZTs. The heat of interaction could not be measured for ASA because of the ASA decomposition on the adsorbent surface.

The heats of adsorption obtained by microcalorimetry studies confirm that the adsorption of ATL and SA happens on energetically heterogeneous surfaces. This can be explained by the fact that the studied pharmaceuticals possess electron-donor groups (SA and ASA have O-donor groups (–OH and –OOH), and ATL has both –NH and –OH) and can interact with M^2+^ cations (which are electron acceptors) in different ways depending on the nature of M as well as on the steric requirements imposed by their crystallographic positions in the zeolite lattice [26].

## 4. Use of ZT in Agriculture

### 4.1. Use of ZT and Fe-ZT as Soil Supplements

Natural zeolites have good adsorptive and ion-exchange qualities, making them suitable for agricultural use. They have been viewed as a promising option for a soil supplement that can enhance soil’s chemical and physical characteristics, including their ability to retain water and acidity. This quality is especially crucial for sandy soils [75]. One of the primary advantages of adding ZT to soils is its capacity to extend plant nutrient retention, which prevents nutrient leaching and helps preserve water [76,77,78]. Furthermore, ZT has successfully remediated heavy-metal-polluted soils [79,80,81].

**Figure 3:**
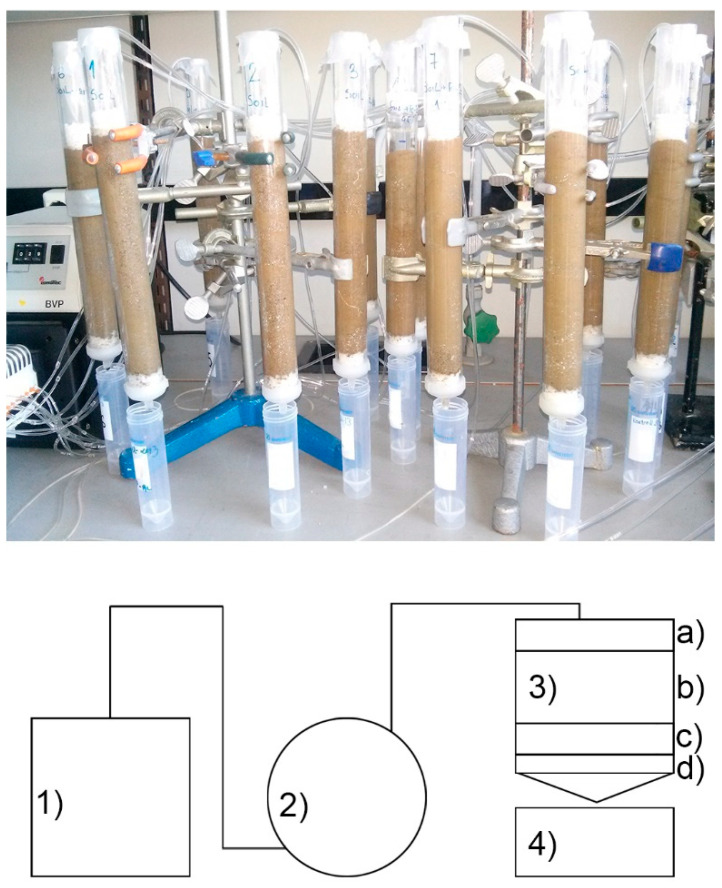
(a) Experimental setup used in the leaching experiments and (b) schematic presentation of an individual column: (1) water tank; (2) peristaltic pump; (3) plexiglass column; (a) PVC balls; (b) soil or soil/Fe-ZT mixture; (c) PVC balls; (d) PVC filter; (4) sample collector.

ZT and Fe-ZT were tested as soil supplements for preserving plant nutrients (nitrogen and potassium) in different soil types: sandy, silty loam, and silty clay soils [13]. This was performed by leaching experiments performed in laboratory conditions using column systems at room temperature (Figure 3).

KNO_3_ was used as a mineral fertilizer. Adding KNO_3_ corresponded to 10 mg N (100 g soil)^−1^ and 28 mg K (100 g soil)^−1^, which amounts to 200 kg N and 550 kg K ha^−1^ soil.

Both ZT and Fe-ZT were active in soils, retaining the nutrients. Their retention efficiency depends on the soil type. Nitrate ions readily leach out, irrespective of the soil type. The high leaching was detected at the beginning of the experiment, and then the leaching proceeded slowly. This is ascribed to the high solubility of nitrate. Silty clay and sandy soils treated with chabazite (Italy) likewise exhibited a high nitrate leaching rate at the start of the leaching experiments [82]. Adding ZT and Fe-ZT increases nitrate retention in silty loam and clay soils. The retention effect is more pronounced for Fe-ZT (42% for Fe-ZT and 26% for ZT) in silty loam soil in contrast to silty clay, where Fe-ZT retains only 14% of N (ZT efficiency was 4%).

Their physico-chemical properties can explain the difference in retention activity in different soil types. Silty clay soil is alkaline, and it seems likely that there is a competition between the nitrate and hydroxyl ions for the adsorption sites on the zeolite, causing leaching of the nitrate.

Adding both ZT and Fe-ZT improves the retention of potassium in the studied soils in the following order: silty loam < silty clay << sandy soil. The highest retention was evident for sandy soil, which is important since sandy soils are poor in clay content and have limited ability to bind potassium. This effect is minor for the silty loam and clay soils since both can retain potassium [13].

The results confirm the applicability of ZT and Fe-ZT as soil supplements for retaining plant nutrients (nitrogen and potassium) in different soil types. On sandy soils, the addition has the most noticeable effect. Similarly, research demonstrates that K^+^ leaching can be considerably reduced even in sandy soil supplemented with municipal compost [83]. It has also been reported that K-enriched ZT can be utilized to stop the loss of K^+^ from soil when applied to sandy soil and sandy soil that has been supplemented with chemical fertilizers [84].

### 4.2. Use of the Se-Containing Fe-ZT for the Growth of Pleurotus Ostreatus

To obtain SeO_3_-Fe-ZT and SeO_4_-Fe-ZT, Fe-ZT was treated with water solutions of Na_2_SeO_3_ (4.5 mmol dm^−3^) and Na_2_SeO_4_ (3.0 mmol dm^−3^) at pH = 8 and pH = 3, respectively [14]. The Se XANES spectra of SeO_3_-Fe-ZT confirmed the presence of selenite (37%) and selenate (63%), which were found in SeO_4_-Fe-ZT. The Se K-edge EXAFS analysis provides insight into the local structure around the Se atoms in both Se-containing products. In the case of SeO_3_-Fe-ZT, it is suggested that the selenite adsorption involves the formation of both Se–O–Fe and Se–O–Si bonds, while in the case of SeO_4_-Fe-ZT, adsorption primarily occurs through the formation of Se–O–Fe bonds.

Both products containing selenium were utilized to cultivate *P. ostreatus* mycelia. The Se content in mushrooms cultivated on the substrate with SeO_3_-Fe-ZT was about 211 μg g^−1^ and lower than in mushrooms grown on the substrate with SeO_4_-Fe-ZT (~254 μg g^−1^). The presence of Se in mushrooms shows that *P. ostreatus* converts inorganic Se into an organically bound form by adsorbing it. The amount of Se in the utilized substrate is determined by its oxidation number. The cultivation of *P. ostreatus* onto a substrate mixed with the Se-containing ZT is a promising method for obtaining Se-enriched dietary supplements since the latter have been known to possess antioxidant activity [15].

### 4.3. Catalytic Use of ZT

One of the main concerns in the engineering of the atmospheric environment is the removal of NOx. NOx gases are typically produced when fuels burn at high temperatures. One of the methods for reducing NOx that has received the greatest attention is the selective catalytic reduction of NOx (SCR-NOx). According to Moreno-Tost et al. [85], clinoptilolite has demonstrated excellent performance as a catalyst for the selective catalytic reduction of NOx by ammonia (NH_3_-SCR). The propane-SCR-NOx process has studied the catalytic efficacy of clinoptilolite exchanged with transition metal ions (Zn^2+^, Fe^2+^, Cu^2+^, and Mn^2+^). The Cu- and Mn-ZT performed the best conversion rate. The concentration of strong acidic sites, redox centers, highly specific surface areas, and additional framework species were found to be responsible for the conversion activity [86].

At ZT, wet impregnation and calcination at 500 °C were used to deposit oxides of cobalt, manganese, and mixed cobalt-manganese oxides. Using the XPS technique, Mn^3+^ and Mn^4+^ ions were found. Co_3_O_4_ is produced in both bi-component Co-Mn-ZT and Mn-ZT Mn-ZT, whereas MnO_2_ is produced at Mn-ZT. On the ZT surface, the active phases were uniformly distributed and highly dispersed, leading to complete oxidation of the n-hexane. The sample containing the highest concentration of Co^3+^ ions exhibited the highest catalytic activity [87].

ZT was modified with hydrochloric acid, iron, copper, and cobalt salts to obtain catalytic properties for converting dihydroxyacetone (DHA) based biomass. During the conversion of DHA, lactic acid, formic acid, pyruvic acid, acetic acid, and levulinic acid were obtained. The highest lactic acid yield (66.2%) was achieved with Co-ZT, formic acid (93.6%) with Cu-H-ZT, and 87.4% acetic acid with Fe-ZT. The catalytic activity was proposed for a partial dealumination of clinoptilolite lattice and reduction of the Fe and Cu species [88].

A partial dealuminated ZT from deposit Kučin (Slovakia) was tested in the liquid phase isomerization of *α*-pinene. The results indicate that the catalytic activity of ZT is an entangled function of chemical composition, crystallinity, overall acidity, and substrate access to the active sites [5].

ZT purchased from Sepifeed (Turkey) was catalytically active in the isomerization of geraniol under mild conditions. Thumbergol, used in cancer treatment, was obtained at 47 mol.% [89].

The catalytic performance of NiO-ZT, Cu_2_O-ZT, and Zn-ZT was studied in the pyrolysis of hardwood lignin using a bench-scale fixed-bed reactor [12]. The catalysts were mixed with lignin and then treated under an N_2_ atmosphere at 500 °C. GC and GC/MS were used to analyze the resulting pyrolysis gas and liquid products.

Synergistic interaction between the ZT lattice and nano oxide particles explains the bio-oil production with high phenol content. The type of nano oxide present in ZT impacts the yield of phenols, with NiO-ZT exhibiting the highest value (approx. 54%). In the presence of NiO-ZT, the yield of unwanted oxygenated compounds (esters, carbonyls, and organic acids) decreases. Still, harmful polycyclic aromatic hydrocarbons (PAHs) do not rise significantly. This indicates that the clinoptilolite lattice’s microporous constrictions do not favor the side-effect catalytic reactions that produce PAHs.

The results lead to the conclusion that the pyrolysis of hardwood lignin cannot be related to the acidity of the catalysts. A significant number of Lewis acid sites were found for ZnO-ZT, but the phenol content decreased compared to Na-ZT. In contrast, Cu_2_O-ZT possesses many Lewis acid sites, increasing the bio-oil phenol content [12].

The results show that the clinoptilolite-based catalysts can have a significant role in the catalytic pyrolysis of lignin to bio-oils.

TiO_2_-ZT was obtained using TiCl_4_ and partially dealuminated ZT at a high temperature. The oxide form of Ti-immobilized on dealuminated clinoptilolite was formed in the anatase phase, with the zeolite structure remaining intact, according to powder XRD and EDS analyses. This catalyst’s activity was tested in the esterification of 1-octanol with acetic acid. It was suggested that firstly, acetic acid interacts with the surface hydroxyl groups of the catalyst with intermolecular forces (H-bonding), and then the alcohol reacts with the adsorbed acid molecule to form the alkyl acetate product with condensation [90].

Levulinic acid (LA) is one of the platform chemicals obtained from biomass, and its esterification to levulinate esters is an industrially important process. The esterification is an acid-catalyzed reaction for which strong, harmful sulfuric acid is used. Many efforts have been made to substitute for sulfuric acid. The catalytic activity of SnO_2_-ZT and SO_4_-SnO_2_-ZT was tested in the LA esterification with ethanol (EOL) and octanol (OOL) [17].

The obtained catalytic results show that both SnO_2_-ZT and SO_4_-SnO_2_-ZT are catalytically active in the LA esterification with both alcohols. SnO_2_-ZT exhibits high activity (around 55%) in converting LA to octyl levulinate (OLE). The catalytic activity is not affected by the Sn content. That can be due to hindered access of the long chain of octanol to all acid sites, especially those inside the pores of the clinoptilolite lattice. Significantly lower catalytic activity is achieved in converting LA to ethyl levulinate (ELE). The conversion rate increases with the Sn content (up to 22%), indicating that the catalytic active sites on the catalysts become more accessible to smaller ethanol molecules.

**Figure 4:**
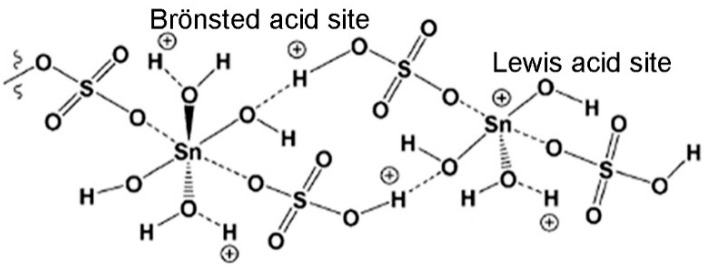
Schematic presentation of Lewis and Brönsted acid sites onto SO_4_-SnO_2_-ZT.

A total conversion of LA to both OLE and ELE was accomplished by SO_4_-SnO_2_-ZT, which is explained by the acidity of the samples, i.e., the presence of high amounts of both the Brönsted and Lewis acid sites (Figure 4). It is concluded that sulfate groups significantly influence the esterification reaction, supported by reported data [91,92].

Reusability tests revealed that the process by which LA is converted to esters differs for EOL and OOL. Because of the length of the chain, it appears that the esterification reaction with OOL mostly happens at the catalyst’s exterior surface. On the other hand, because EOL molecules can penetrate the catalyst’s pores more deeply, there is less diffusion out of the intermediate products and ELE, which increases coke formation and reduces activity. Additionally, reusability tests revealed that (a) the first partial leaching of sulfate groups from the catalysts under test reduces the extent of the LA conversion and (b) the extent of the LA conversion is stabilized following the second cycle, indicating that structural characteristics of the clinoptilolite lattice prevent further leaching [18].

When all the data are considered, it seems that the clinoptilolite lattice can limit the extent of the sulfate leaching, providing reusable catalysts and preventing the aggregation of catalytically active SO_4_-SnO_2_ particles. This increases dispersion and improves accessibility for the reactants.

#### Photocatalytic Activity

Heterogeneous photocatalysis has emerged as the most widely studied advanced oxidation process (AOP). Suspensions of powdered TiO_2_ in the treated solution have been used in most studies on photocatalytic degradation of organic pollutants [93]. However, several characteristics make it difficult to fully utilize TiO_2_’s photo efficiency, including poor adsorption, low surface area, the absorption of only a small portion of sunlight, rapid recombination of electron–hole pairs, and difficulty separating from solution. Several attempts have been made to circumvent this limitation to improve the efficiency of photocatalysts using suitable supports. Zeolites have been widely used among various supports due to their unique structural properties.

ZT belongs to the Semnan region (Iran) was used to prepare NiO-containing zeolite [94]. NiO-ZT has an essential role in the photodegradation of aqueous cefalexin (degradation efficiency was 73.5%), while pure NiO and zeolite did not have significant photodegradation efficiency [94]. Moreover, photocatalytic degradation of a 4-nitrophenol aqueous solution was investigated using ZnO-nano-ZT under UV irradiation. The photocatalyst was prepared by ion-exchanging nano-ZT in a zinc nitrate aqueous solution for 24 h, followed by calcination at 450 °C for 12 h. The results indicated that the photodegradation rate was affected by the initial 4-nitrophenol concentration, the pH, and the amount of catalyst [95].

CuO-ZT prepared via wet impregnation of parent zeolite with CuSO_4_ aqueous solution and calcination was reported as an efficient photocatalyst in the degradation of p-aminophenol under sunlight irradiation. As for ZnO-ZT, it was concluded that the ZT host was important in the photodegradation process, so pure CuO and natural zeolite did not have significant photodegradation efficiency [96].

NiS-ZT can effectively degrade furfural exposed to UV light. Selecting the optimal parameters to accelerate the rate of degradation is crucial. Since it is outside the zeolite framework and does not exhibit a significant degradation efficiency, the zeolite lattice plays a crucial role in the degradation process, indicating that the active centers within the zeolite structure are NiS. One significant benefit of photodegradation is that very little photocatalyst (330 mg dm^−3^) is used, which conserves photocatalyst, conserves photons because of the decreased scattering, and ultimately reduces environmental contamination [97].

ZT is suitable for photocatalytically active metal oxide particles such as SnO_2_, TiO_2_, ZnO, NiO, and CuO [98,99,100,101,102]. An enrichment in their catalytic activity was attributed to a synergistic effect between the particles of metal oxides and lattice of ZT. ZT prevents the aggregation of metal oxide particles by fixing them onto ion-exchange sites and enables electron–hole recombination.

Photocatalytic efficacy of SnO_2_-ZT and ZT was evaluated in the degradation of methylene blue (MB), a representative cationic dye. Besides ZT, several zeolitic tuffs from deposits in different regions (Turkey, Iran, Romania, and Slovakia) were tested for comparison [14,16]. Using a batch reactor system, photocatalytic tests were carried out at room temperature and atmospheric pressure and under visible light irradiation.

SnO_2_-ZT is photocatalytically active, increasing both the adsorption capacity and photocatalytic performance, which can be attributed to a high surface area and a partial increase in the negative potential of the surface. The degradation of MB is affected by the Sn content. Increasing the Sn content above an optimal amount decreases its photocatalytic activity under visible light illumination. This is attributed to the SnO_2_ aggregation, a decrease in the effective surface area, and the collision of SnO_2_ particles with free MB molecules.

It can be proposed how ZT functions in photocatalytic systems as present in this study. The synergistic effect of the clinoptilolite lattice and SnO_2_ particles is responsible for the photolytic activity. Because of ZT’s adsorption affinity for cationic organic dyes, more molecules are drawn to the catalyst surface, where the produced hydroxyl radicals create many active sites for the adsorption of intermediates. Additionally, the lattice inhibits their aggregation by anchoring the SnO_2_ particles to specific crystallographic sites while simultaneously permitting electron–hole recombination.

ZT also exhibits exceptional photocatalytic activity in the MB degradation process. pH impacts the overall degradation of MB, peaking at pH = 6 (70% for *C*_0_ = 10 mg MB dm^−3^, 0.2 g ZT dm^−3^, for 300 min). The photodegradation follows the kinetic model of Langmuir–Hinshelwood. The Fe species, typically found as impurities in zeolitic tuffs, have a combined effect on the initial adsorption and degradation of MB upon exposure to visible light, which is responsible for the entire degradation of the dye. Zeolitic tuffs from other regions give similar results. The activity rises with the tuffs’ Fe content. Additionally, pure ZT from a deposit in Ukraine showed photocatalytic activity in the rhodamine B degradation due to iron impurities in tuff [103].

### 4.4. Interactions of Bacteria and ZT

Bacteria’s interaction with zeolite depends on its chemical characteristics (native or modified ZT). The characteristics determine the type of zeolite-bacterium interactions, which can then influence the target species of bacteria (e.g., useful or pathogenic bacteria). As a model, the useful bacterium *Acinetobacter junii*, which is used in tertiary-stage wastewater treatment, is chosen. Besides the widely tested *Escherichia coli* and *Staphylococcus aureus*, an emerging hospital pathogen, *Acinetobacter baumannii*, is also chosen as a model pathogenic bacterium.

In a water column, bacteria have a native tendency to attach to the solid surface, where they continue to grow in the form of biofilm. The bacterial population in the biofilm, opposite to the planktonic population, will be protected from different environmental biotic and abiotic stresses [27]. The inorganic surface should be inert, nontoxic, of porous structure, relatively cheap, easily available, and environmentally friendly and provide a rough, irregular surface for bacterial colonization [104]. ZT meets all the mentioned characteristics to serve as a carrier of immobilized bacteria.

To elucidate the crucial factors determining the extent of bacterial immobilization onto different ZTs of the same particle size, ZTs of the particle size ~0.125 mm originating from Croatia, Turkey, and Serbia were examined [27]. After 24 h of contact, immobilized *A. junii* was in the order of a few billion bacteria per gram of dry ZT. The extent of bacterial immobilization could not be correlated with the main ZT features, such as the clinoptilolite content, cation-exchange capacity, or zeta potential. The extent of bacterial immobilization on a single ZT cannot be predicted by mineralogical and chemical analysis of ZT. The results conclude that each ZT is a candidate to be used as a carrier of bacteria, with the only limitation that ZT must not have a high concentration of toxic heavy metals.

In contact with bacteria suspended in water with ZT, the attachment of bacteria on the surface of the ZT will appear within one hour. The intensive immobilization of ZT (billions of bacteria per gram of ZT) is achieved after 12 h of contact, and prolonged contact until 24 h will ensure stable bacterial biofilm [27].

The rate of bacterial immobilization depends more on the features of the material and, to a lesser extent, on the features of the bacterium. The structure of the bacterial cell wall, widely classified as Gram-positive or Gram-negative, will not determine the immobilization. For the attachment to solid surfaces, bacteria use the structures on the cell surface that are pili or capsules. A hydrophilic (having pili at the surface) and hydrophobic (having capsule on the surface) isolate of *A. baumannii* was used to elucidate the effect of the hydrophobicity level of the bacterial surface on the intensity of immobilization onto the hydrophilic surface of ZT [28]. Both *A. baumannii*, with hydrophilic or hydrophobic cell surfaces, were immobilized onto ZT (5.2 and 6.9 log CFU (Colony Forming Units)/g, respectively) in the form of biofilm within 24 h of contact.

**Figure 5:**
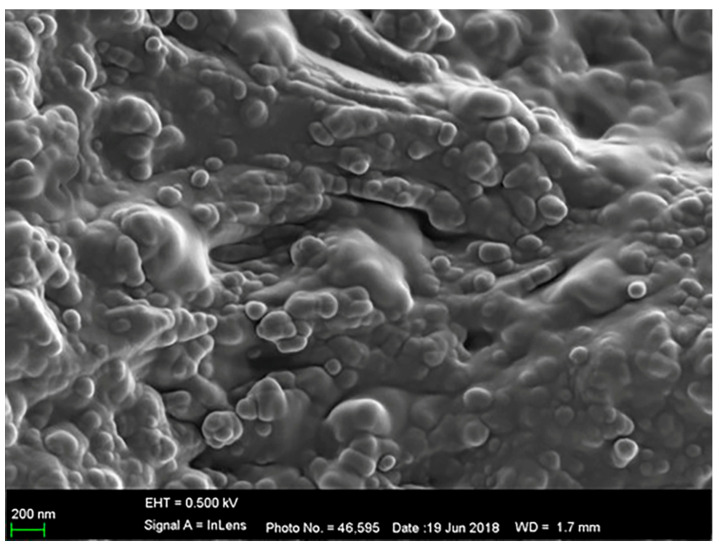
Cells of *A. baumannii* immobilized onto the ZT particles covered with extracellular polymer.

The nutrient availability for heterotrophic bacteria influences bacterial survival and multiplication in water. The immobilization of *A. baumannii* onto ZT in nutrient-poor and nutrient-rich water was followed for 72 h [28]. Planktonic cells of *A. baumannii* present in water became quickly attached to the surface of ZT particles, regardless of the nutrient concentration in water. Immobilized cells excrete the extracellular polymers and form stable biofilm on the particles within 24 h (Figure 5). No further incorporation of planktonic cells occurs in the formed biofilm.

**Figure 6:**
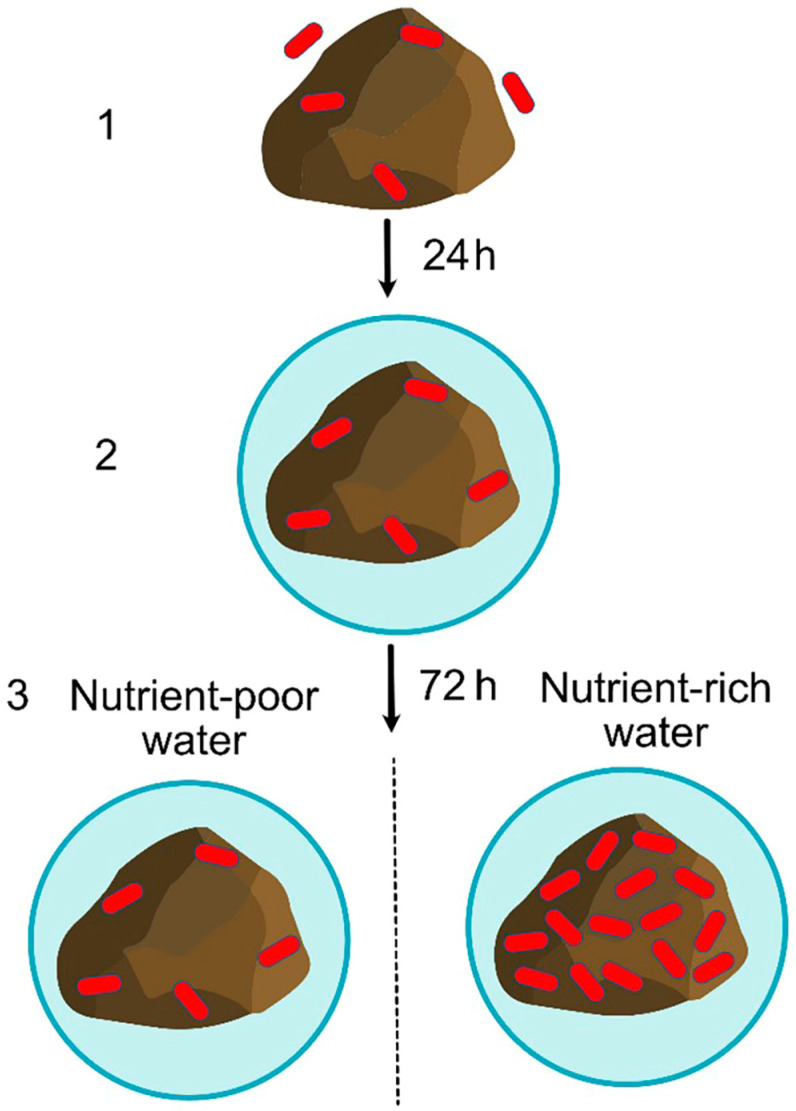
Mode of bacterial immobilization onto ZT. (1) Planktonic cells are quickly attached to the surface of ZT particles; (2) immobilized cells excrete the extracellular polymers and form biofilm; (3) in the nutrient-poor water, biofilm stays conserved, while in the nutrient-rich water, the number of bacteria in biofilm increases.

In nutrient-poor water, the shortage of nutrients prevents the multiplication of bacteria previously incorporated in the biofilm; consequently, the biofilm stays conserved. In nutrient-rich water, the availability of nutrients enables the multiplication of bacteria inside the initially formed biofilm, which increases the number of bacteria and further matures the biofilm. According to the behavior of bacteria in nutrient-poor and nutrient-rich water media, the mode of *A. baumannii* immobilization onto ZT is proposed, whereby this mode can be applied to the other bacterial species (Figure 6).

Another parameter that influences the extent of bacterial immobilization is the particle size of ZT. The extent of bacterial immobilization increases with the decrease in particle size of ZT [30]. Under the same conditions, immobilized *A. junii* decreased from 9.7 to 8.5 CFU/g when aparticle size ˂ 0.125 mm or 0.5–1.0 mm was used, respectively.

#### 4.4.1. Possible Applications of Bacterial Immobilization onto ZT

As bacteria can be easily immobilized onto ZT particles, ZT can be used as a carrier of beneficial bacteria. Bioparticles consisting of phosphate-accumulating bacteria *A. junii* immobilized onto ZT incorporate very well into activated sludge biomass [31]. The bioaugmentation of activated sludge with 5 g dm^−3^ of bioparticles significantly improved the phosphorus removal from fresh municipal wastewater. Better phosphorus removal is a function of increased biomass of phosphate-accumulating bacteria *A. junii*.

Bacteria immobilized onto ZT particles are protected from the grazing of ciliates and rotifers in the activated sludge and protected from being washed away by large amounts of wastewater [27]. Applying ZT in the tertiary stage of wastewater treatment can be used as a low-cost, efficient, and energy-saving technique to improve phosphorus removal from wastewater.

#### 4.4.2. Interactions of Bacteria and M–ZT

The ZT containing heavy metals or bactericidal organic substances was studied to eliminate unwanted pathogenic bacteria [30]. The antibacterial effect of such modified ZT depends on the type and concentration of the modified ZT used, the chemical composition of the water medium, the concentration and species of bacteria, and contact time with bacteria. The modified ZTs act bactericidally by the direct contact of particles with bacteria or via the leaching of biologically toxic ions.

The antibacterial activity of Cu-ZT, Zn-ZT, or Ni-ZT at the concentration of 1 wt.% was tested against Gram-negative bacterium *E. coli* and Gram-positive bacterium *S. aureus* at the initial bacterial concentration of 7 log CFU/mL in different water media after short (1 h) and long-term (24 h) exposure [32]. The Ni-ZT showed weak antibacterial activity as compared to Cu-ZT and Zn-ZT. Antibacterial activities of the modified ZTs in a nutrient-rich medium were significantly lower than those in the nutrient-depleted media. The increased antibacterial activity of modified ZTs was evident by prolonging the contact time with bacteria. There was no remarkable difference in the antibacterial activity against Gram-negative and Gram-positive bacteria. The antibacterial activity of modified ZTs depends more on the features of the material and, to a lesser extent, on those of the bacterium.

The antibacterial activity of the Cu_2_O, ZnO, or NiO nanoparticles supported on ZT was investigated in the secondary effluent wastewater [33]. The Cu_2_O- and ZnO-containing ZT are more effective than the NiO-containing ZT, comparable to Cu-ZT, and Zn-ZT compared to Ni-ZT. After 24 h of contact, the Cu_2_O and ZnO-containing ZT reduced the numbers of *E. coli* and *S. aureus* by four to six orders of magnitude. In the real wastewater, the bactericidal effect (meaning 100% reduction) against native *E. coli* was obtained after 1 h of contact. An increase in the NiO-ZT concentration from 1 to 5 wt.% did not enhance the antibacterial activity against *E. coli*. This fact suggests that the antibacterial activity of nano oxide particles supported on ZT depends on the chemical nature of the toxic metal and that an increase in the ZT content could not increase the antibacterial activity.

The Ag-ZT, Cu-ZT, and benzalkonium-containing ZT (BC-ZT) showed bactericidal activity against clinical isolates of *A. baumannii* in a physiological solution, depending on the type and concentration of ZTs used and the contact time [34]. Differences between the bacterial isolates only slightly influenced the bactericidal activity. Minimum bactericidal concentrations (MBC) after 24 h of contact varied from 31 to 250 mg dm^−3^, 125 to 250 mg dm^−3^, and 250 to 500 mg dm^−3^ for Ag-ZT, Cu-ZT, and BC-ZT, respectively.

Ag-ZT showed a superior bactericidal effect compared to Cu-ZT or Zn-ZT against isolates of *E. coli* [35]. Ag-ZT showed a bactericidal effect within 1 h of contact in a nutrient-rich medium, while Cu-ZT or Zn-ZT were bactericidal only in nutrient-poor water media.

Except in water media, Ag-ZT and Cu-ZT were tested for the elimination of *A. baumannii* from artificially contaminated natural soils [36]. Adding 1wt.% of Cu-ZT shortened the survival of *A. baumannii* from seven to three days in slightly acidic terra rossa and from four months to 14 days in slightly alkaline red palaeosol.

Adding 0.1 wt.% of Ag-ZT to slightly acidic or alkaline soil completely removed the *A. baumannii* within 1 h of contact, with a negligible impact on native soil bacteria.

Salycilate-containing BC-ZT at a concentration of 0.5 wt.% [37] immediately killed all *E. coli* and *S. aureus* in a nutrient-rich medium. The bactericidal effect was ascribed to the benzalkonium cations, while the salicylate anions had a negligible effect on the bacteria. It was shown that the arrangement of the BC layers onto ZT is crucial in the antibacterial activity of BC-ZT [3]. BC-ZT with the BC bilayer or patchy BC bilayer coverage shows a bactericidal effect. In the case of monolayer coverage, an antibacterial effect is expected, while in partial monolayer coverage, bacteria are unaffected.

Forming a biofilm on abiotic surfaces is one important virulence factor of *A. baumannii*. Novel composites with anti-biofouling activity were developed to prevent biofilm formation on the tubes for medical applications [38]. Ag-ZT was added to a polyvinyl chloride matrix, followed by coating with D-Tyrosine. Composites containing 1 wt.% of Ag-ZT showed a bactericidal effect against the clinical isolate of *A. baumannii* in the phosphate-buffered saline within 24 h of contact.

#### 4.4.3. Other Possible Applications of M-ZT 

The antibacterial activity of metal-containing ZT can be used to disinfect water containing low concentrations of nutrients, such as drinking water or secondary effluent wastewater. An M-ZT with low leaching of metal cations, such as Cu- or Cu_2_O-containing ZT [32,33], is recommended for disinfection. The bactericidal activity of ZT with Cu(II), Ag(I), or BC can be applied in the disinfection of water or wet surfaces [34] but also in the remediation of soils contaminated with human pathogens [36].

ZTs can be used in medicine except for environmental applications. The BC-ZT and salicylate-containing BC-ZT [37] can be an alternative drug with simultaneous antibacterial and anti-inflammatory effects. Composite material containing the Ag-ZT is promising in preventing the biofilm formation of *A. baumannii* on tubes used for medical applications [38].

## 5. Conclusions

This review summarizes the results of extensive studies on tuffs rich in clinoptilolite from the deposits in Serbia and other countries. Because clinoptilolite is present there in high content, the tuffs can be recommended in wastewater treatments to adsorb and remove a wide range of inorganic (such as heavy metal cations, oxyanions, nitrates, and phosphates) and organic (such as organic micropollutants) pollutants from water.

Clinoptilolite’s thermal and chemical stability makes it useful for creating ultrafine, catalytically active oxides or as a carrier of catalytically active species. Its catalytic activity has been confirmed in several catalytic processes.

Additionally, clinoptilolite is a beneficial addition to the soil since it improves the soil’s nutritional qualities and can supply certain elements that it lacks, like selenium.

In conclusion, clinoptilolite is a potential antibacterial agent and a carrier of beneficial bacteria for the biotechnological treatment of activated sludge and wastewater.

This suggests that natural clinoptilolite has considerable potential for use as an environmentally friendly material in various applications, including synthetic zeolites that have long been regarded as superior.

## Figures and Tables

**Table 1:** Removal efficiency (%) of Na-ZT toward M(II) at the initial concentration of 1.5 mmol M(II) dm^−3^ at 25 °C.

M(II)	Mg	Mn	Ni	Cu	Zn	Pb
Removal, %	60	47	15	84	50	100

## Data Availability

Not applicable.
